# Sesamol Alleviates the Cytotoxic Effect of Cyclophosphamide on Normal Human Lung WI-38 Cells via Suppressing RAGE/NF-κB/Autophagy Signaling

**DOI:** 10.1007/s13659-020-00286-6

**Published:** 2020-11-20

**Authors:** Soad Z. El-Emam

**Affiliations:** Department of Pharmacology and Toxicology, Faculty of Pharmacy, October 6 University, 6 October City, Giza, 12566 Egypt

**Keywords:** Cyclophosphamide, Sesamol, Lung toxicity, Autophagy, Oxidative stress, RAGE

## Abstract

**Abstract:**

Cyclophosphamide (CYL) is a chemotherapeutic medication commonly used in managing various malignancies like breast cancer or leukemia. Though, CYL has been documented to induce lung toxicity. Mechanism of CYL toxicity is through oxidative stress and the release of damage-associated molecular patterns (DAMPs). Sesamol (SES) is a natural antioxidant isolated from *Sesamum indicum* and its effect against CYL-induced lung toxicity is not studied yet. This study aims to investigate whether SES could prevent any deleterious effects induced by CYL on lung using normal human lung cells, WI-38 cell line, without suppressing its efficacy. Cells were pretreated with SES and/or CYL for 24 h, then cell viability was estimated by MTS and trypan blue assays. The mode of cell death was determined by AO/EB staining. Additionally, caspase-3 level, oxidative stress, and inflammatory markers were evaluated by colorimetric and ELISA techniques. qRT-PCR was performed to evaluate RAGE, NF-κB, and Beclin-1 mRNA-expression. CYL-treated WI-38 cells developed a significantly increased cell death with enhanced oxidative and RAGE/NF-κb/Autophagy signaling, which were all attenuated after pretreatment with SES. Thus, we concluded that SES offered a protective role against CYL-induced lung injury via suppressing oxidative stress and RAGE/NF-κB/Autophagy signaling, which is a natural safe therapeutic option against CYL toxicities.

**Graphic Abstract:**

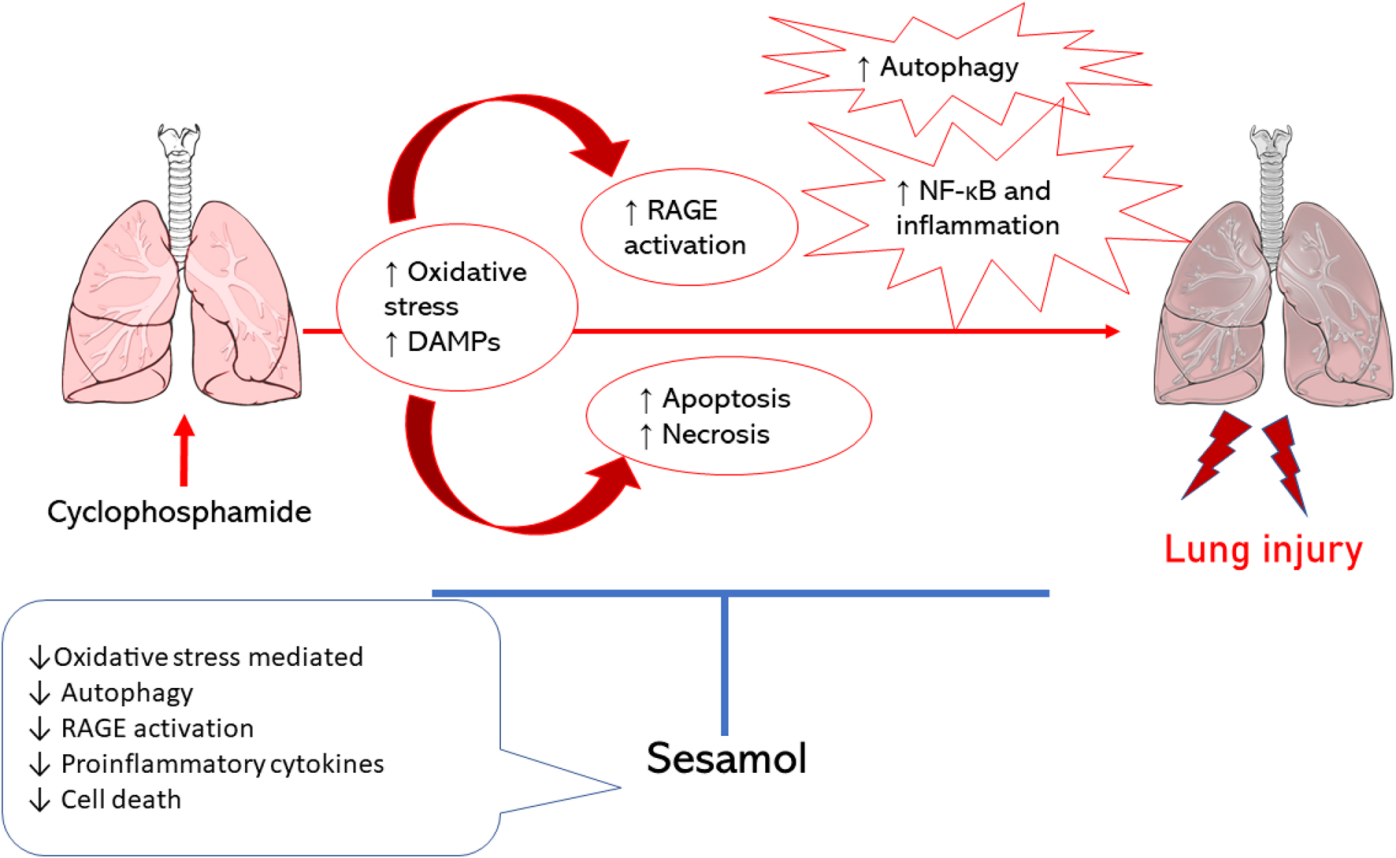

**Electronic supplementary material:**

The online version of this article (10.1007/s13659-020-00286-6) contains supplementary material, which is available to authorized users.

## Introduction

Cyclophosphamide (CYL) is one of the commonly chemotherapeutic alkylating agents used in treating different types of malignancies including solid tumors and hematological malignancies in addition to treating several autoimmune diseases due to its immunosuppressive properties [1]. However, its therapeutic use is limited due to the associated organ toxicities as cardiotoxicity [2], renal [3] and hepatic toxicities [4] in addition to lung toxicity [5]. CYL is metabolized by cytochrome P450 isozyme to aldophosphamide which is further decomposed by aldehyde dehydrogenase to phosphoramide mustard and acrolein. Phosphoramide is the active anticancer agent that acts on seven-guanine residues of DNA resulting in cancer cell death, whereas CYL-induced toxicity is in consequence of the formation of acrolein toxic metabolite [6, 7]. Additionally, High mobility group box 1 (HMGB1), one of damage-associated molecular patterns (DAMPs), plays an essential role in activating inflammatory signaling due to CYL as a result of activating the receptor for advanced glycation end-products (RAGE) [8, 9].

RAGE signaling pathway has a crucial role in acute lung injury through increasing the levels of proinflammatory cytokines via RAGE-mediated nuclear factor-kappa B (NF-κB) activation [10]. Additionally, it was reported that RAGE-ligand binding triggers an autophagic response to oxidative stress [11]. Autophagy, a type II programmed cell death, is activated in stressful conditions such as starvation and exposure to chemotherapeutic drugs [12]. It is a dynamic process controlled by autophagy-related genes (Atg) to maintain normal cellular homeostasis by removing damaged organelles and proteins. Among the Atg, Beclin 1, which is a main regulator of autophagy and it has a role in the formation of autophagosomes [13]. After autophagy induction, the cytosolic form of the microtubule-associated protein light chain 3 (LC3)-I is conjugated to form LC3-II as an early autophagy marker [14].

Sesamol (SES) is a natural antioxidant isolated from sesame seeds (*Sesamum indicum*) and sesame oil. Its therapeutic ability has been intensively explored, and there is convincing evidence that SES functions as a metabolic agent of antioxidant, anti-mutagenic, anti-hepatotoxic, anti-inflammatory, anti-aging, and chemopreventive effects in addition to its anticancer properties [15]. SES can be used safely to augment the antioxidant defenses of healthy organs and tissues thus affording protection against chemotherapy-induced organ toxicities which are major side effects [16]. SES effect against CYL-induced lung toxicity has not been studied yet. Therefore, this study aims to investigate the protective effect of SES against CYL-induced lung toxicity without reducing its efficacy on cancer cells and to explore the effect of SES on RAGE/NF-κB/autophagy signaling that is known to be activated by CYL.

## Results

### Cytotoxicity and Cell Viability Assessment

**Fig. 1:**
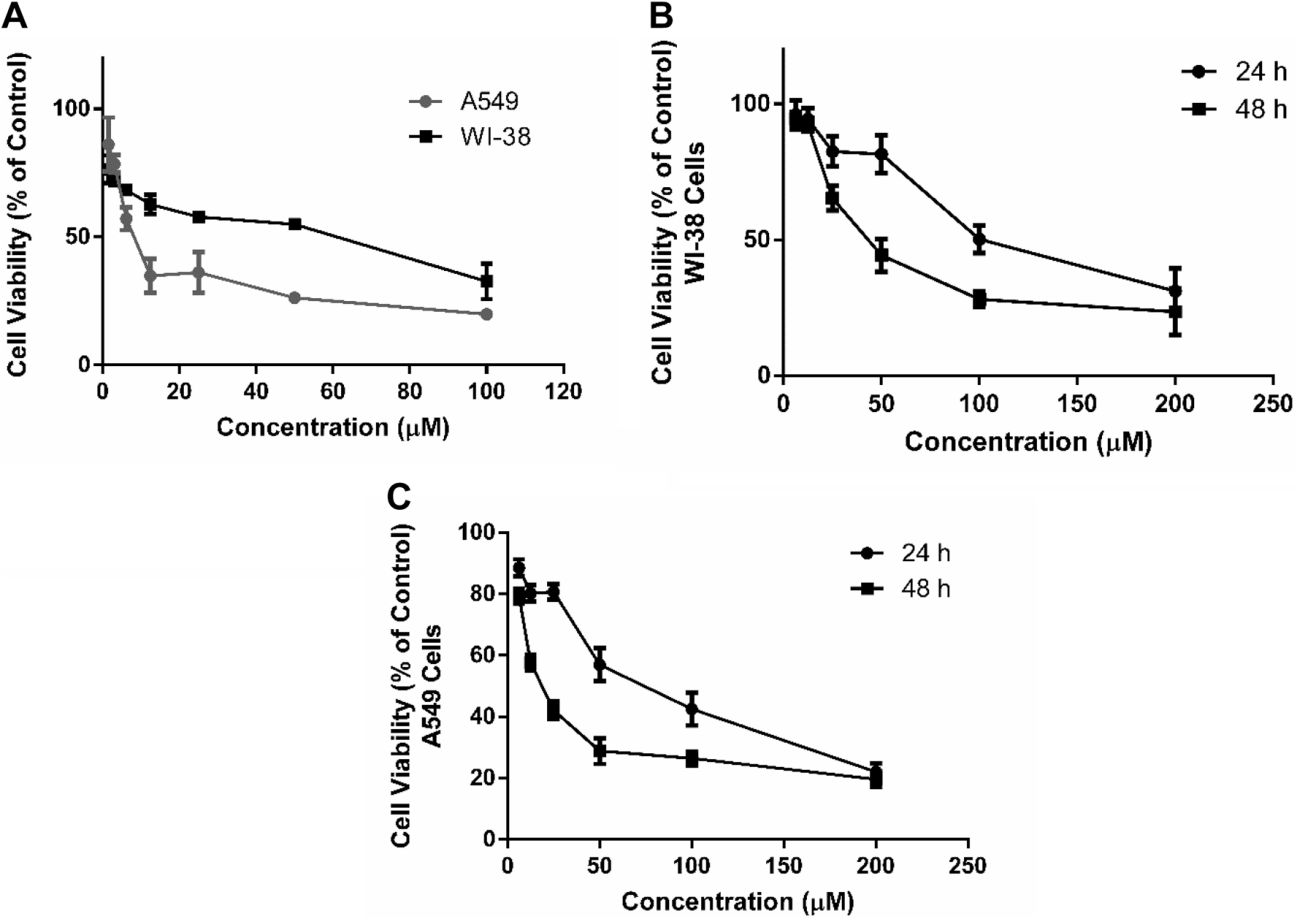
Cell viability assessment by MTS assay. **A** Effect of different concentrations of CYL against percentage of cell viability of WI-38 and A549 cells after 24 h incubation for evaluating IC_50_. **B** Cell viability-response curve of SES in WI-38 cells after incubation for 24 and 48 h for selecting the suitable therapeutic dose. **C** Cell viability response curve of SES in A549 cells after incubation for 24 h and 48 h. The experiments were performed independently in triplicates and data are expressed as Mean ± S.D. (n = 3)

The cytotoxic effect of CYL was evaluated by determining the effect of different concentrations of CYL (100–1.56 µM) on the viability of WI-38 and A549 cells after 24 h of incubation using MTS assay. As presented in Fig. 1A, the cell viability was decreased by increasing the concentration of CYL when compared with untreated cells (control group) and the half-maximal inhibitory concentrations (IC_50_) were calculated to be 40.7 µM and 10.5 µM respectively for both cell lines. On the other hand, to select the suitable dose for SES, cell viability was also evaluated by MTS assay after treating WI-38 and A549 cells with different concentrations of SES (200–6.25 µM) and incubated for 24 and 48 h. According to SES concentration-cell viability response curve, the IC_50_ was estimated after 24 h and 48 h of incubation to be 110.4 µM and 48.9 µM respectively for WI-38 cells (Fig. 1B) and 69 µM and 21 µM respectively for A549 cells (Fig. 1C). Accordingly, the concentration of 12.5 µM of SES was selected to study its protective effect against CYL-induced lung toxicity, based on using the highest dose with maximal cell viability when compared to the control group.

### SES Pre-treatment Attenuates CYL-Induced Cytotoxicity in Normal Lung Cells

**Fig. 2:**
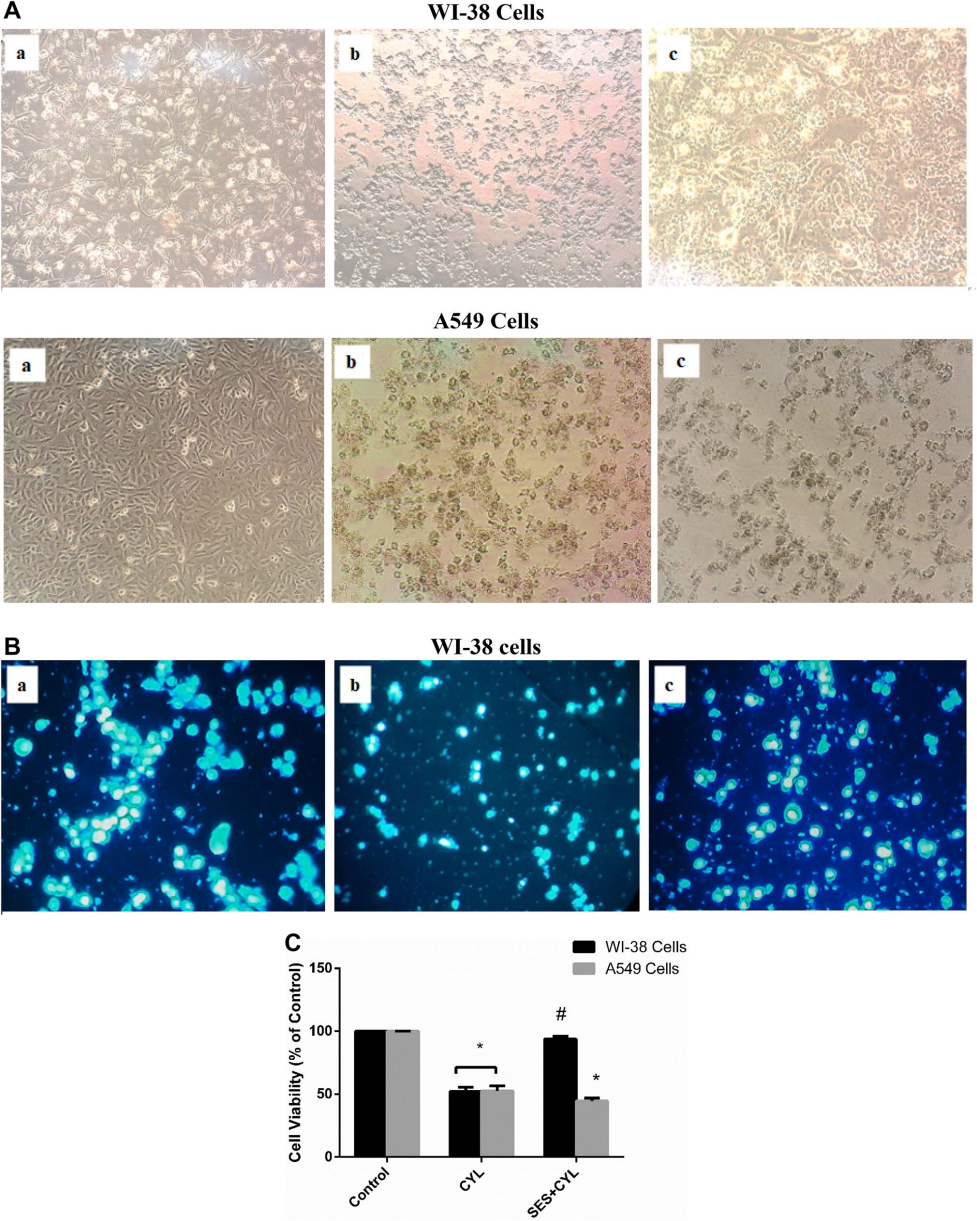
The protective effect of SES against CYL-induced cytotoxicity in normal lung cells. **A** The morphological change induced by CYL in WI-38 cells and A549 cells; (a) Control group; (b) CYL-treated group; (c) SES + CYL- treated group. **B** The effect of CYL on nuclear condensation in WI-38 cells observed by fluorescence microscope using DAPI staining; (a) Control group; (b) CYL-treated group; (c) SES + CYL- treated group. **C** The percentage of cell viability by trypan exclusion test showing the protective effect of SES against CYL-induced cell death in WI-38 cells. The experiments were performed independently in triplicates and data are expressed as Mean ± S.D. (n = 3), *p < 0.05 when compared with control group. #p < 0.05 when compared with CYL group. *CYL* cyclophosphamide, *SES* sesamol

Normal WI-38 cells are characterized by their spindle-like shape with smooth edges, exposing WI-38 cells to CYL (40.7 µM) for 24 h changed cells morphology with inducing cell shrinkage and abnormal shape with nuclear condensation observed on DAPI staining (Fig. 2A and B). To evaluate the extent of SES effect on CYL efficacy against cancer cells, the change in cellular morphology was assessed for A549 cells. As demonstrated in Fig. 2A, A549 cells treated with CYL alone or with SES pretreatment showed changed morphology with aberrant appearance when compared with the control cells.

By trypan exclusion assay, pretreatment of WI-38 cells with SES 12.5 µM 24 h before CYL, significantly attenuated CYL-induced cytotoxicity and cell viability was significantly improved by 1.8 folds when compared with that treated with CYL only (Fig. 2C). While, the percentage of cell death induced by CYL in A549 cells was not diminished by SES pretreatment, thus, the protective effect of SES against CYL was obvious in normal cells rather than cancer cells.

### SES Halts Apoptotic and Necrotic Cell Death Induced by CYL

**Fig. 3:**
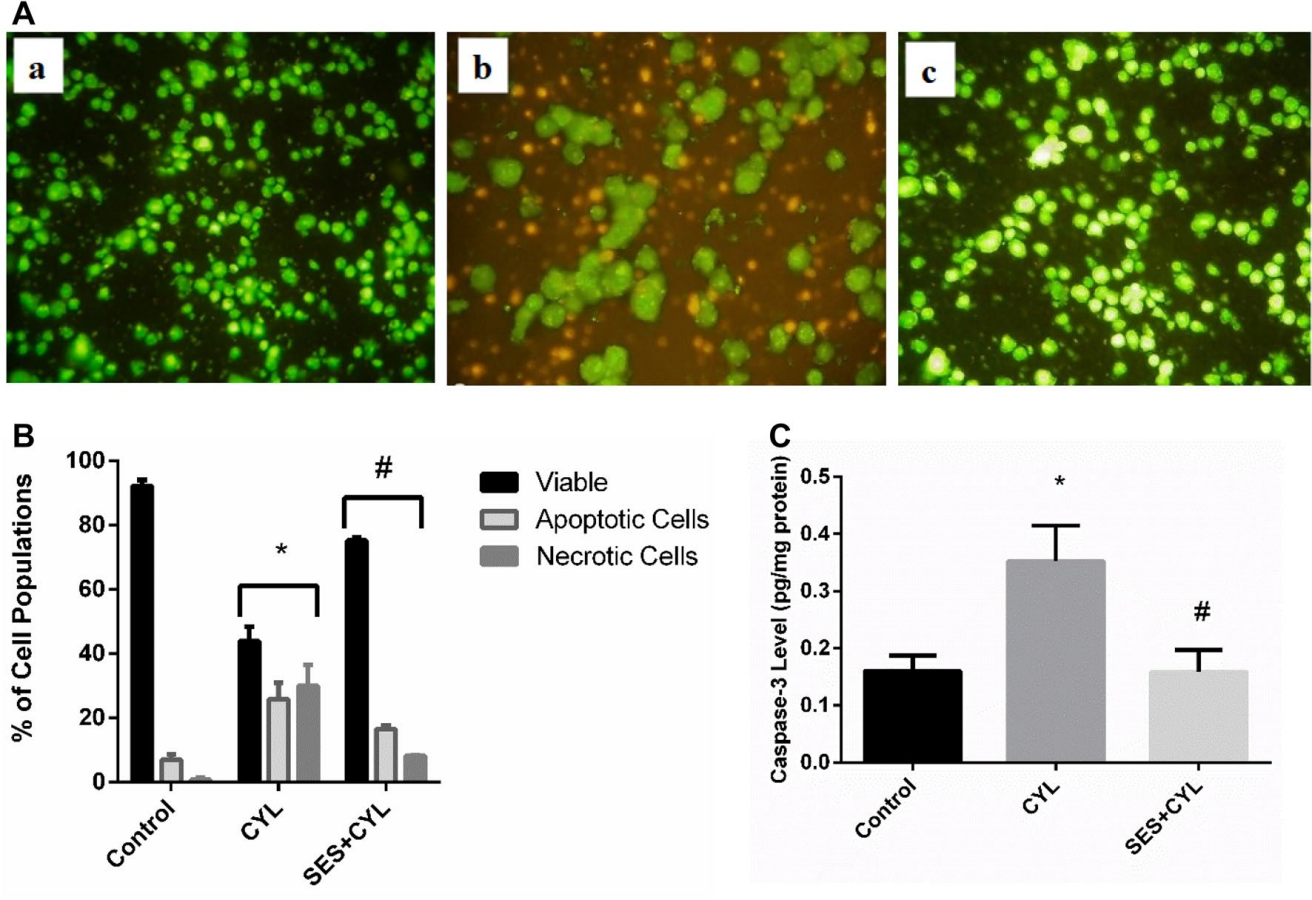
SES effect on CYL-induced apoptotic and necrotic cell death in WI-38 cells. **A** AO/EB fluorescent stain for evaluating mode of cell death in WI-38 cells: live (green), Apoptotic Cells (yellow and light orange), necrotic cells (dark orange and red) staining imaged by a fluorescent microscope a: Control group; b: CYL-group; c: SES + CYL group. **B** The percentage of different cell populations, viable, apoptotic and necrotic cells. **C** Caspase-3 level in WI-38 cells. The experiments were performed independently in triplicates and data are expressed as Mean ± S.D. (n = 3), *p < 0.05 when compared with control group. #p < 0.05 when compared with CYL group. *CYL* cyclophosphamide, *SES* sesamol

CYL reduced viable cells significantly by about 50% and increased both apoptotic and necrotic cell populations as evidenced by AO/EB staining (Fig. 3A and B). Additionally, the caspase-3 level was increased in CYL-treated cells when compared with the control group (p < 0.05) indicating the activation of the apoptotic mode of cell death in WI-38 cells by CYL in addition to necrosis (Fig. 3C). However, SES pretreatment preserved viable WI-38 cells and reduced both apoptotic and necrotic cell populations as well as normalized caspase-3 levels as compared with the CYL group (p < 0.05).

### SES Reduces Oxidative Stress Induced by CYL in Lung Cells

**Fig. 4:**
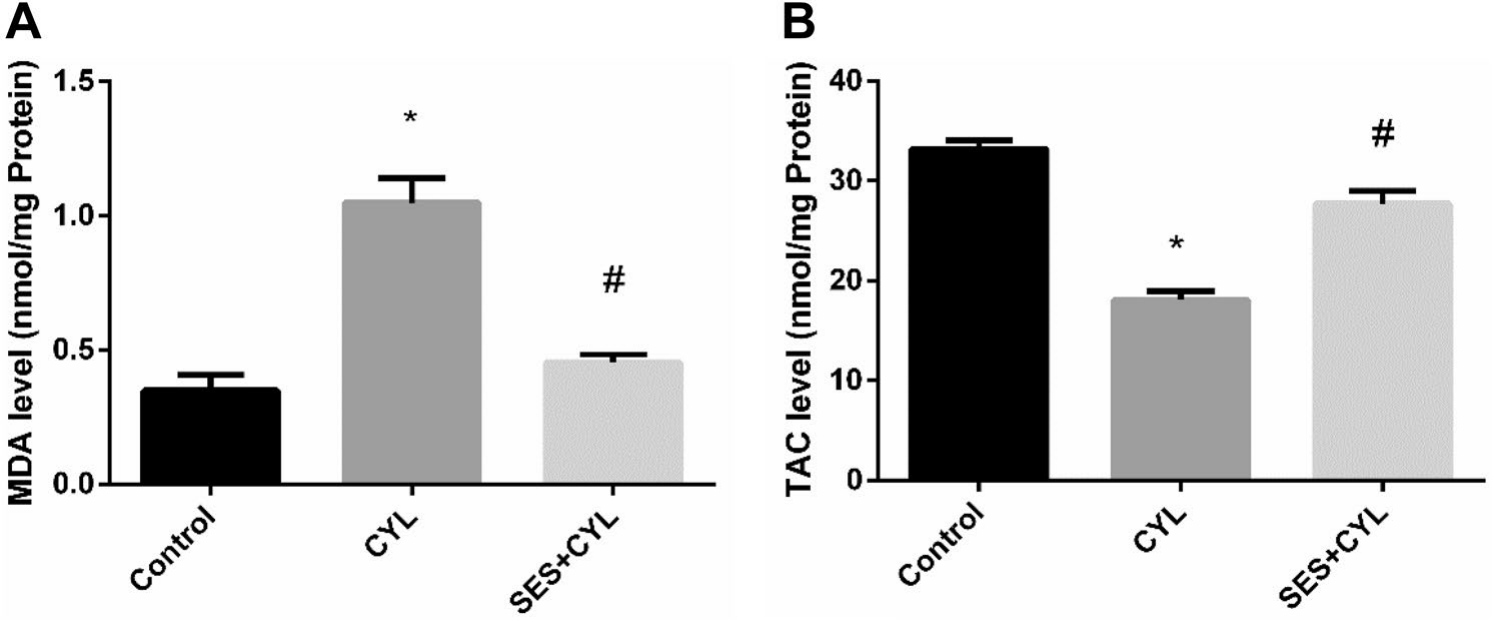
SES effect on oxidative stress induced by CYL in WI-38 cells. **A** MDA Level in WI-38 cells. **B** TAC level in WI-38 cells. The experiments were performed independently in triplicates and data are expressed as Mean ± S.D. (n = 3), *p < 0.05 when compared with control group. #p < 0.05 when compared with CYL group. *CYL* cyclophosphamide, *SES* sesamol

CYL enhanced oxidative stress significantly by increasing the level of MDA, which is a marker of lipid peroxidation, by double folds and reducing the level of TAC by 54% when compared with the control group (p < 0.05). The oxidant/antioxidant balance was restored in WI-38 cells by pretreatment with SES when compared with the CYL group (p < 0.05), as presented in Fig. 4.

### SES Modulates the Inflammatory Response Induced by CYL in Lung Cells

**Fig. 5:**
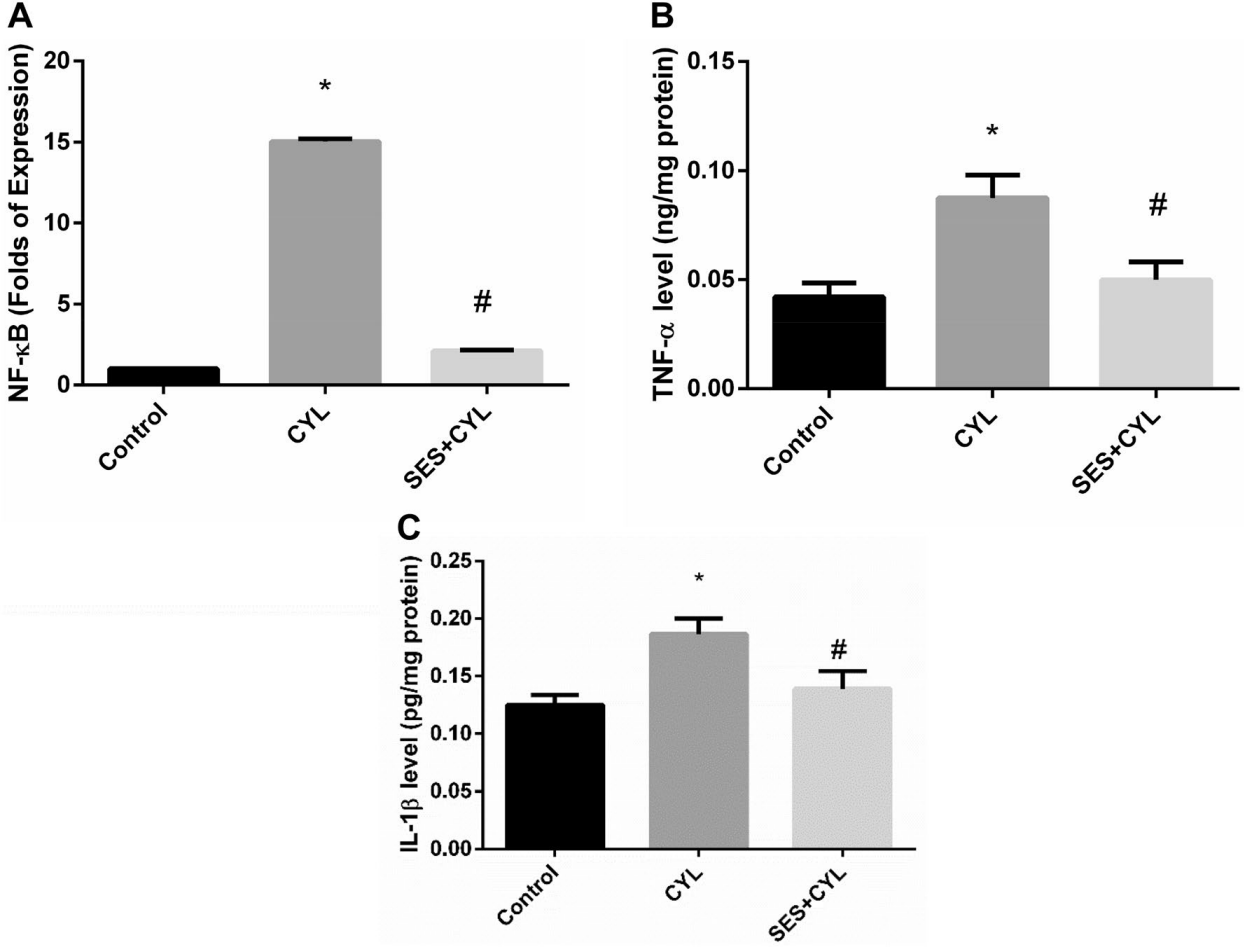
SES suppressed the inflammatory cytokines induced by CYL treatment. **A** The level of NF-κB expression in WI-38 cells. **B** The level of TNF-α in WI-38 cells. **C** The level of IL-1β in WI-38 cells. The experiments were performed independently in triplicates and data are expressed as Mean ± S.D. (n = 3), *p < 0.05 when compared with control group. #p < 0.05 when compared with CYL group. *CYL* cyclophosphamide, *SES* sesamol

As shown in Fig. 5, CYL induced inflammatory responses by increasing the expression of NF-κB by 15 folds as well as increasing the levels of TNF-α and IL-1β in WI-38 cells as compared with the control group (p < 0.05); however, pretreatment with SES showed a significant anti-inflammatory effects by reverting the expression levels of the inflammatory cytokines as compared with the CYL group (p < 0.05).

### SES Attenuates RAGE and Autophagy Signaling Induced by CYL in Lung Cells

**Fig. 6:**
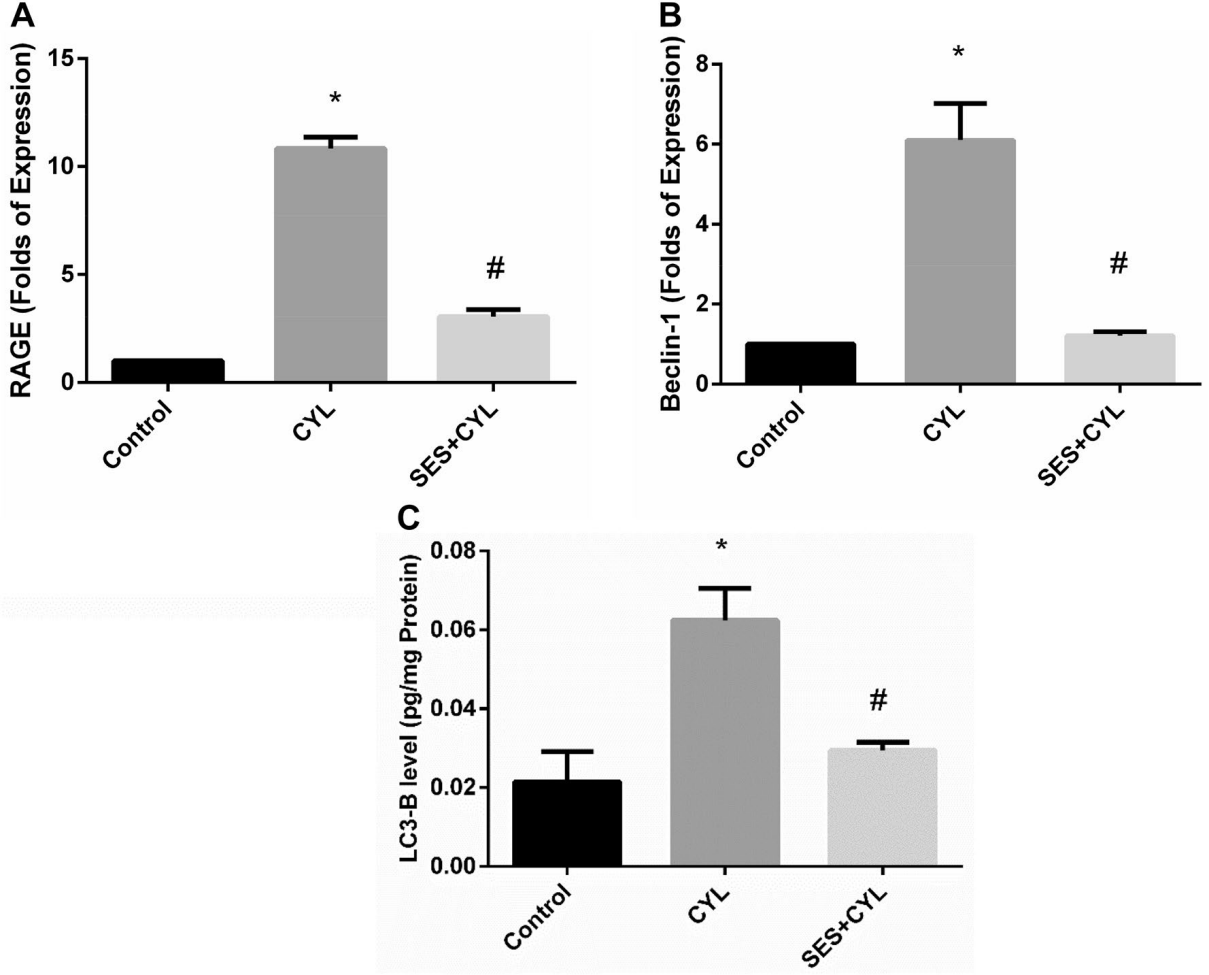
SES ameliorated CYL induced RAGE/autophagy signaling activation. **A** The expression level of RAGE in WI-38 cells. **B** The expression level of Beclin-1 in WI-38 cells. **C** The level of LC3-B in in WI-38 cells. The experiments were performed independently in triplicates and data are expressed as Mean ± S.D. (n = 3), *p < 0.05 when compared with control group. #p < 0.05 when compared with CYL group. *CYL* cyclophosphamide, *SES* sesamol

Treatment of WI-38 cells with CYL significantly increased the expression of RAGE and increased autophagy signaling evidenced by increased Beclin-1 expression and LC3-B level when compared with the control group (p < 0.05). Alternatively, cells pretreated with SES showed suppressed expression of RAGE and reduced autophagy signaling when compared with the CYL group (p < 0.05) as presented in Fig. 6.

## Discussion

Nowadays, there is a great concern about using natural compounds to alleviate the side-effects of chemotherapy resulting from ROS generation and oxidant injury in cellular systems [17]. SES is a dietary phytochemical isolated from sesame, several studies have reported its antioxidant and anti-inflammatory effects; since Duarte et al. reported that SES pretreatment for an hour afforded protection against mitochondrial impairment in lipopolysaccharide-treated RAW 264.7 cells [18]. In the same vein, our study showed the protective effect of SES against CYL-induced cytotoxicity in normal cells rather than cancer cells through ameliorating CYL-induced oxidative stress by increasing TAC and reducing MDA levels.

CYL is an alkylating agent widely used as chemotherapy for treating numerous neoplastic diseases [5]. Additionally, CYL is used as an immunosuppressive drug in the management of various auto-immune diseases like rheumatoid arthritis [19]. Lung toxicity is one of CYL common dose-limiting and life-threatening adverse event owing to the formation of reactive metabolites that are responsible for peroxidative injury of lung cells; thus, the manifestation of CYL-induced lung injury [6].

As presented in our results, treatment of WI-38 cells with CYL changed the oxidative conditions by inducing lipid peroxidation and reducing the level of TAC resulting in oxidative stress. This is in accordance with several studies including that of Şengül et al. [20], who reported that ROS generation is dependent on acrolein related CYL which inhibits the antioxidant system thus mediating the pro-inflammatory responses. Chen et al. [21] have reported that CYL-induced inflammation is mediated by DAMPs including HMGB1, heat shock protein 60 (HSP60) and glucose-regulated protein 94 (Grp94) resulting in organ damage. DAMPs are released from dead cells in response to cellular stress or injury, inducing inflammatory responses and their release is considered as a marker for tissue damage [22]. This could explain the increased expression of RAGE in CYL-treated WI-38 cells in the current study. RAGE, a transmembrane receptor, is overexpressed by different types of cells when associated with several ligands, including advanced glycation end product (AGEs) and DAMPs in response to cells exposed to oxidative stress, then activating the inflammatory cascade [23, 24]. The initiation of RAGE signaling contributes to chronic inflammatory disorders through activating NF-κB and releasing inflammatory cytokines [25, 26]. In this study, we demonstrated the protective effect of SES against the inflammatory responses induced by CYL by repressing the expression of NF-κB and hence the inflammatory cytokines release as TNF-α and IL-1β. This is supported by other previous investigations showing that SES attenuates systemic LPS-induced lung inflammation by inhibiting the inflammatory response via supressing NF-κB activation and TNF-α release [27]. Additionally, Yashaswini et al. reported that SES inhibits the activation of inflammatory oxygenase, lipoxygenase, due to its ability to scavenge hydroperoxides [28].

Concerning autophagy in the current study, CYL enhanced the expression of Beclin-1 and increased the level of LC3-B protein in WI-38 cells. This is in line with the results of Kang et al. who reported that CYL activates apoptosis and autophagy signaling resulting in acute renal injury [29]. Autophagy signaling activation is considered as a defense system since the key intracellular signal transducers of autophagy are ROS generation and oxidative stress signaling activation [30, 31]. Along the same lines, the study of Zhao showed that activation of autophagy plays a protective role in CYL-induced cystitis by reducing inflammation and oxidative stress [32]. The current study demonstrated the ability of SES to suppress autophagy triggered by oxidative stress due to CYL treatment in WI-38 cells via decreasing Beclin-1 expression and LC3-B protein level. This is consistent with Tang et al. who reported that the use of antioxidants inhibits oxidative stress-induced autophagy as demonstrated in animal and human cell lines models [11].

Besides the role of RAGE signaling in activating the inflammatory cascade via enhancing NF-κB nuclear translocation, it also mediates autophagy which participates in the pathogenesis of different lung diseases including acute lung injury [33]. Therefore, decreasing DAMPs release and inhibiting the interaction of RAGE with its ligands is a possible treatment strategy for CYL-induced lung injury.

In conclusion, SES has a protective effect on normal lung cells against CYL-induced injury without affecting its efficacy on cancer cells. Our results illustrated the antioxidant and the anti-inflammatory effects of SES are due to suppressing RAGE/ NF-κB/autophagy signaling; therefore, SES can be a natural safe therapeutic option against CYL toxicities without defeating its efficacy.

## Experimental Section

### Drugs and Chemicals

Sesamol and all chemicals were purchased from Sigma Aldrich Chemical Co. (St. Louis, MO, USA) except for those stated elsewhere.

### Cell Culture

In this study, we used normal human embryonic lung fibroblast, WI-38 cell line, to study the protective effect of SES against CYL-induced injury. Furthermore, to explore whether SES can affect CYL efficacy on cancer cells or not, we used a human lung carcinoma, A549 cell line.

WI-38 and A549 cells were obtained from the American Type Culture Collection (ATCC, USA). Cells were maintained with Dulbecco's Modified Eagle's Medium (DMEM) supplemented with 10% fetal bovine serum (FBS) and 1% v/v penicillin–streptomycin under 37 °C with 5% CO_2_ in a humidified incubator.

### MTS Assay

To estimate the used concentrations of SES and CYL in our experiment on WI-38 and A549 cells, MTS assay was performed according to the manufacturer’s instructions (Abcam, USA). In brief, cells (1 × 10^4^ cells/well) were seeded in 96-well culture plates with DMEM medium supplemented with 10% FBS and incubated overnight at 37 °C with 5% CO_2_ in humidified incubator to allow cells to settle down; then cells were treated with various concentrations for each drug and then incubated for 24 h or 48 h. After incubation time, MTS reagent 20 μl were added to each well and incubated for further 4 h at 37 °C and then the absorbance was measured using the microplate ELISA reader (FLUOstar Omega, BMG, Labtech, Germany) at 490 nm, The absorbance of the resulting color is directly proportional to the number of the viable cells in each sample. The percentage of relative cell viability was calculated using the following equation: [Absorbance of treated cells/ Absorbance of control cells)] × 100. The IC_50_ was determined by using a program Graph-Pad PRISM version 6.

### Samples Preparation

In six-well culture plates, WI-38 or A549 cells were seeded with DMEM media supplemented with 10% FBS at 37 °C with 5% CO_2_ in a humidified incubator. The wells were grouped as the following (three wells for each group): Control group: cells were incubated in complete DMEM media only without any drug treatment. CYL group: cells were treated with CYL using the IC_50_ value estimated by MTS assay then incubated for 24 h. SES + CYL group: cells were pretreated with SES 12.5 µM for 24 h then treated with CYL using a fresh media and then incubated for further 24 h. The dose of SES was selected according to the cell viability MTS assay by using the most effective and maximal safe dose on WI-38 cells.

After the incubation time, samples were prepared in three sets and each set was repeated three times individually. In the first set, cells were collected by trypsinization, washed twice with PBS, and used directly for trypan blue assay and acridine orange/ethidium bromide fluorescent stain. The second set, cells were collected by scraping, washed twice by ice-cold PBS and the cell pellets were lysed in ice-cold lysis buffer supplemented with protease inhibitor cocktail and the cells were passed through a 21-gauge needle, then centrifuged at 14,000×*g* for 15 min at 4 °C and the supernatants were used for estimating lipid peroxidation, TAC and ELISA. The third set cells were collected by scraping and used directly for mRNA extraction for quantifying gene expression of NF-κB, Beclin-1, and RAGE.

### Trypan Blue Assay

To evaluate the effect of SES against CYL-induced cytotoxicity in WI-38 and A549 cells, trypan blue assay was performed by adding 10 µl of trypan blue 0.4% to an equal volume of cell suspension and mixed well then both dead and viable cells were counted under a light microscope using a hemocytometer [34]. The percentage of viability was calculated by the following equation: % of cell viability = (number of viable cells/total cell number) × 100.

### DAPI Nuclear Staining

4′,6-diamidino-2-phenylindole (DAPI) stain was used to assess nuclear morphology of WI-38 cells [35]. Directly after harvesting control and treated cells, cells were rinsed with PBS, and incubated with 4′,6- DAPI 1 μM for 15 min at 37 °C. after incubation, cells were rinsed then photographed using a fluorescent microscope (Olympus, Japan).

### Acridine Orange/Ethidium Bromide Fluorescent Stain

Mode of cell death was evaluated by determining apoptosis and necrosis ratio using acridine orange/ethidium bromide (AO/EB) fluorescent staining. Collected WI-38 cells were stained using a dye mixture of AO and EB 1:1 in PBS, then were examined using fluorescence microscopy within 20 min [36]. For each sample, at least 500 cells were counted, and the percentage of apoptotic or necrotic cells was calculated as follows: % of apoptotic or necrotic cells = (total number of apoptotic or necrotic cells/total number of cells counted) × 100.

### Enzyme-Linked Immunosorbent Assay (ELISA)

ELISA was used for estimating the levels of inflammatory cytokines as TNF-α and IL-1β in cell supernatant, in addition to estimating caspase-3 and LC3-B levels in cell lysate, according to the manufacturer’s protocols of the ELISA kits (Sunlong Biotech, China).

### Evaluating Antioxidant/Oxidative Stress Markers

Total antioxidant capacity (TAC) and lipid peroxidation were estimated in cell lysates for all groups. The end product of lipid peroxidation, malondialdehyde (MDA), was detected spectrophotometrically at 534 nm using a commercial kit (Biodiagnostic Co., Cairo, Egypt). TAC was estimated in Trolox equivalents at 570 nm according to the manufacturer’s instructions using the TAC assay kit (sigma-Aldrich, USA).

### Quantitative Real Time-PCR (qRT-PCR)

The gene expression magnitude for RAGE, NF-κB, and Beclin was quantified using RT-PCR. Briefly, total RNA was extracted from the collected cells using RNeasy Mini kit (Qiagen, Germany, GmbH) following the manufacturer's instructions. The primers (Metabion, Germany), listed in Table 1, were utilized in a 25 µl reaction containing 12.5 µl of the 2 × QuantiTect SYBR Green PCR Master Mix (Qiagen, Germany, GmbH), 0.25 µl of RevertAid Reverse Transcriptase (200 U/µL) (Thermo Fisher), 8.25 µl of water, and 3 µl of RNA template. The reaction was performed in a Stratagene MX3005P real-time PCR machine. Amplification curves and ct values were determined by the stratagene MX3005P software. To estimate the variation of gene expression of different samples, the ct of each sample was compared with that of the positive control group using the following ratio: (2^−∆∆ct^). GAPDH was used as the housekeeping gene.

**Table 1:** The list of oligonucleotide sequences of the gene-specific primers used in qRT-PCR

Gene	Sequence (5′–3′)
GAPDH	F: CTCTGATTTGGTCGTATTGGG R: TGGAAGATGGTGATGGGATT
Beclin1	F: GGCTGAGAGACTGGATCAGG R: CTGCGTCTGGGCATAACG
NF-KB	F: TGGTGCCTCACTGCTAACT R: GGATGCACTTCAGCTTCTGT
RAGE	F: CTGATCCTCCCACAGAGCC R: CAGGACCAGGGAACCTACAG

### Statistical Analysis

The statistical analysis and the graphical presentation of the data were performed using Prism software version 6.01 (GraphPad Software Inc., CA, USA). Data were presented as mean ± standard deviation. Statistical significance was determined using one-way ANOVA following with a Tukey–Kramer post hoc test for evaluating the differences between groups. The statistically significant level was established at p-value < 0.05.

## Electronic supplementary material

Below is the link to the electronic supplementary material.

Electronic supplementary material 1 (DOCX 1040 kb)

## Abbreviations

CYL: Cyclophosphamide
HMGB1: High mobility group box 1
DAMPs: Damage-associated molecular patterns
RAGE: Receptor for advanced glycation end-products
NF-κB: Nuclear factor-kappaB
Atg: Autophagy-related genes
LC 3: Microtubule-associated protein light chain 3
SES: Sesamol
