# Supplementary material for: Sesamol Alleviates the Cytotoxic Effect of Cyclophosphamide on Normal Human Lung WI-38 Cells via Suppressing RAGE/NF-κB/Autophagy Signaling
Source: Nat Prod Bioprospect. 2020 Nov 20;11(3):333–43. doi: 10.1007/s13659-020-00286-6 (PMC8141072; doi:10.1007/s13659-020-00286-6)
Supplement: Supplementary file 1 — Electronic supplementary material 1 (DOCX 1040 kb) [file 13659_2020_286_MOESM1_ESM.docx]

**Supplemental Original Source Data**

1. **Cell viability assessment by MTS assay to determine the effect of different concentrations of CYL against percentage of cell viability of WI-38 and A549 cells after 24 h incubation for evaluating IC50.**

| Cell viability (% of control) | | |  |  |
| --- | --- | --- | --- | --- |
| CYL 24 h Incubation | | | | |
| Concentration (µM) | **WI-38** | | **A549** | |
|  | **Mean** | **SD** |  |  |
| 100.00 | 32.68 | 6.93 | 19.89 | 2.14 |
| 50.00 | 54.90 | 1.36 | 26.28 | 2.03 |
| 25.00 | 57.88 | 2.07 | 36.16 | 7.95 |
| 12.50 | 62.77 | 3.69 | 34.81 | 6.60 |
| 6.25 | 68.30 | 1.98 | 57.10 | 4.45 |
| 3.13 | 71.65 | 1.78 | 78.51 | 3.39 |
| 1.56 | 76.37 | 5.45 | 86.18 | 10.48 |

1. **Cell viability assessment for SES by MTS assay at two different incubation times**

| Cell viability (% of control) | | | | |
| --- | --- | --- | --- | --- |
|  | SES 24 h Incubation | | SES 48 h Incubation | |
| Concentration (µM) | **Mean** | **SD** | **Mean** | **SD** |
| 200 | 31.2 | 8.3 | 23.6 | 6.1 |
| 100 | 50.3 | 5.1 | 28.2 | 10.2 |
| 50 | 81.5 | 7.1 | 44.3 | 21.7 |
| 25 | 82.6 | 5.5 | 65.3 | 12.5 |
| 12.5 | 94.4 | 4.1 | 92.3 | 4.0 |
| 6.25 | 96.0 | 5.3 | 94.7 | 1.5 |

Evaluating the optimum therapeutic dose of SES by exposing WI-38 cells to various concentrations and estimating percentage of cell viability after incubation for 24 and 48 h in respect to control cells. The experiments were performed independently in triplicates and data were expressed as Mean ± S.D. (n = 3)

1. **The percentage of cell viability by trypan exclusion test showing the protective effect of SES against CYL-induced cell death.**

| Cell Viability (% of control) | Control | CYL | SES+CYL |
| --- | --- | --- | --- |
| Mean | **100.0** | **52.11^*^** | **93.81^#^** |
| SD | **-** | **3.400** | **2.106** |
| SEM | **-** | **1.700** | **1.053** |

The experiments were performed independently in triplicates and data were expressed as Mean ± S.D. (n = 3), * p < 0.05 when compared to control group. # p < 0.05 when compared to CYL group. CYL: cyclophosphamide, SES: Sesamol.

1. **The percentage of different cell populations, viable, apoptotic and necrotic cells.**

| Cell Population % | Viable | | Apoptotic Cells | | Necrotic Cells | |
| --- | --- | --- | --- | --- | --- | --- |
|  | **Mean** | **SD** | **Mean** | **SD** | **Mean** | **SD** |
| Control | 92.17 | 1.88 | 7.01 | 1.65 | 0.74 | 0.65 |
| CYL | 43.87^*^ | 4.60 | 25.83^*^ | 5.08 | 29.97^*^ | 6.49 |
| SES+CYL | 75.23^#^ | 1.02 | 16.53^#^ | 0.99 | 8.23^#^ | 0.25 |

The experiments were performed independently in triplicates and data were expressed as Mean ± S.D. (n = 3), * p < 0.05 when compared to control group. # p < 0.05 when compared to CYL group. CYL: cyclophosphamide, SES: Sesamol.

1. **Caspase-3 level in WI-38 cells**

| Caspase-3 level (pg/mg protein) | Control | CYL | SES+CYL |
| --- | --- | --- | --- |
| Mean | 0.16 | 0.35^*^ | 0.16^#^ |
| SD | 0.027 | 0.06 | 0.04 |
| SEM | 0.013 | 0.031 | 0.02 |

The experiments were performed independently in triplicates and data were expressed as Mean ± S.D. (n = 3), * p < 0.05 when compared to control group. # p < 0.05 when compared to CYL group. CYL: cyclophosphamide, SES: Sesamol.

1. **MDA Level in WI-38 cells.**

| MDA level (nmol/mg Protein) | Control | CYL | SES+CYL |
| --- | --- | --- | --- |
| Mean | 0.35 | 1.05^*^ | 0.45^#^ |
| SD | 0.06 | 0.09 | 0.03 |
| SEM | 0.03 | 0.05 | 0.01 |

The experiments were performed independently in triplicates and data were expressed as Mean ± S.D. (n = 3), * p < 0.05 when compared to control group. # p < 0.05 when compared to CYL group. CYL: cyclophosphamide, SES: Sesamol.

1. **TAC level in WI-38 cells**

| TAC level (nmol/mg Protein) | Control | CYL | SES+CYL |
| --- | --- | --- | --- |
| Mean | 33.21 | 18.10^*^ | 27.73^#^ |
| SD | 0.88 | 0.86 | 1.27 |
| SEM | 0.44 | 0.43 | 0.64 |

The experiments were performed independently in triplicates and data were expressed as Mean ± S.D. (n = 3), * p < 0.05 when compared to control group. # p < 0.05 when compared to CYL group. CYL: cyclophosphamide, SES: Sesamol.

1. **Determination of NF-κB expression by RT-PCR in WI-38 cells.**

| NF-κB expression | Control | CYL | SES+CYL |
| --- | --- | --- | --- |
| Mean (CT) | 23.62 | 19.58 | 20.01 |
| Mean (Fold Change) | 1.0 | 15.04^*^ | 2.11^#^ |
| SD | - | 0.17 | 0.05 |
| SEM | - | 0.08 | 0.03 |

Data were expressed as Mean ± S.D. (n = 3), * p < 0.05 when compared to control group. # p < 0.05 when compared to CYL group. CYL: cyclophosphamide, SES: Sesamol.


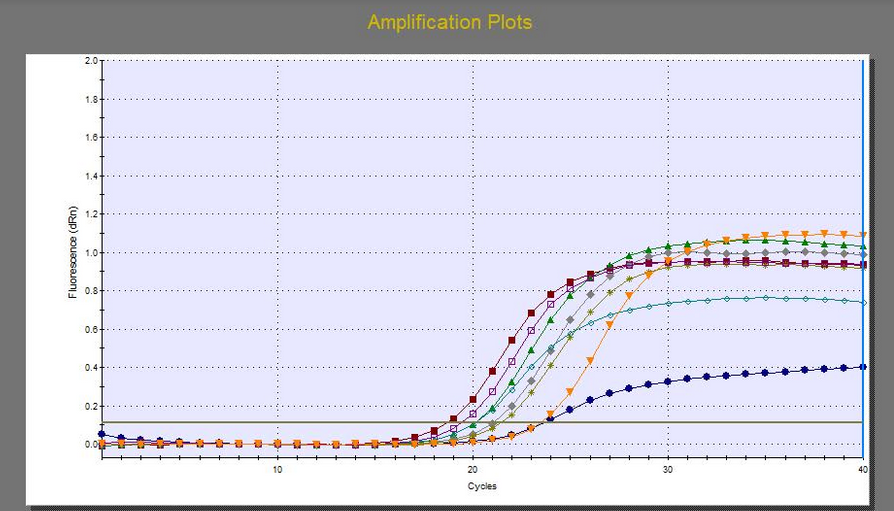


**Amplification plots of NF-κB**

1. **The level of TNF-α in WI-38 cells**

| TNF-α level (pg/mg protein) | Control | CYL | SES+CYL |
| --- | --- | --- | --- |
| Mean | 0.042 | 0.088^*^ | 0.050^#^ |
| SD | 0.010 | 0.011 | 0.008 |
| SEM | 0.003180 | 0.005 | 0.004 |

The experiments were performed independently in triplicates and data were expressed as Mean ± S.D. (n = 3), * p < 0.05 when compared to control group. # p < 0.05 when compared to CYL group. CYL: cyclophosphamide, SES: Sesamol.

1. **The level of IL-1β in WI-38 cells**

| IL-1β level (pg/mg protein) | Control | CYL | SES+CYL |
| --- | --- | --- | --- |
| Mean | 0.13 | 0.19^*^ | 0.14^#^ |
| SD | 0.01 | 0.01 | 0.02 |
| SEM | 0.004 | 0.006 | 0.007 |

The experiments were performed independently in triplicates and data were expressed as Mean ± S.D. (n = 3), * p < 0.05 when compared to control group. # p < 0.05 when compared to CYL group. CYL: cyclophosphamide, SES: Sesamol.

**Determination of RAGE expression by RT-PCR in WI-38 cells.**

| RAGE expression | Control | CYL | SES+CYL |
| --- | --- | --- | --- |
| Mean (CT) | 23.35 | 19.79 | 20.34 |
| Mean (Fold Change) | 1.000 | 10.83^*^ | 3.056^#^ |
| SD | - | 0.5237 | 0.3212 |
| SEM | - | 0.2618 | 0.1606 |

Data were expressed as Mean ± S.D. (n = 3), * p < 0.05 when compared to control group. # p < 0.05 when compared to CYL group. CYL: cyclophosphamide, SES: Sesamol.


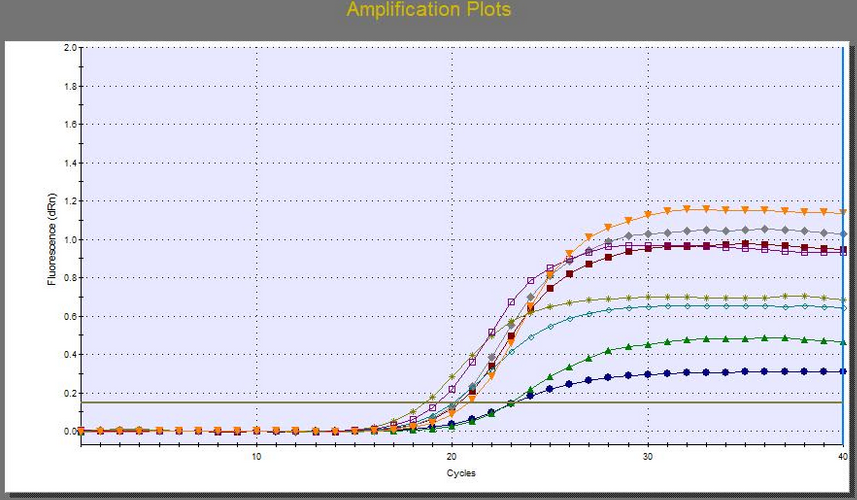


**Amplification plots of RAGE**

**Determination of Beclin-1** **expression by RT-PCR in WI-38 cells.**

| Beclin-1 expression | Control | CYL | SES+CYL |
| --- | --- | --- | --- |
| Mean (CT) | 24.07 | 21.01 | 23.47 |
| Mean (Fold Change) | 1.000 | 6.102^*^ | 1.207^#^ |
| SD | 0.0 | 0.9235 | 0.1051 |
| SEM | 0.0 | 0.4618 | 0.06065 |

Data were expressed as Mean ± S.D. (n = 3), * p < 0.05 when compared to control group. # p < 0.05 when compared to CYL group. CYL: cyclophosphamide, SES: Sesamol.


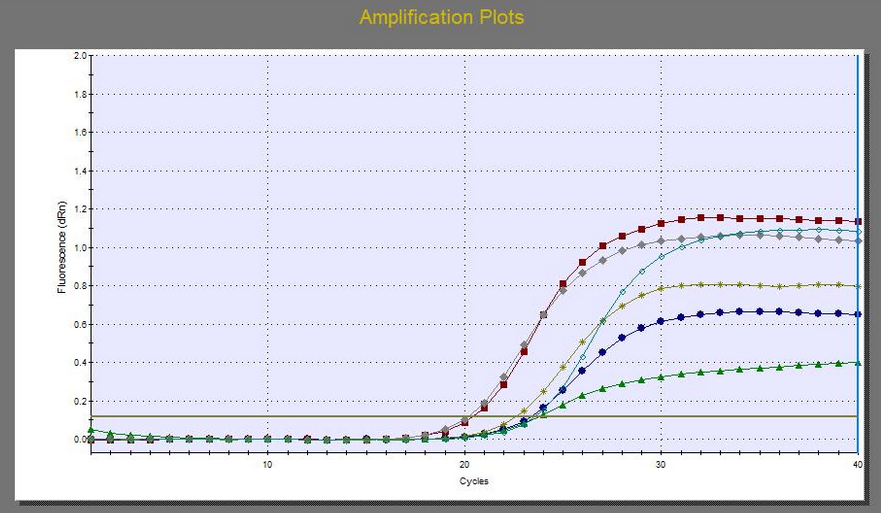


**Amplification plots of Beclin-1**

**The level of LC3-B in in WI-38 cells**

| LC-3 level (pg/mg protein) | Control | CYL | SES+CYL |
| --- | --- | --- | --- |
| Mean | 0.021 | 0.062^*^ | 0.029^#^ |
| SD | 0.008 | 0.008 | 0.002 |
| SEM | 0.004 | 0.004 | 0.001 |

The experiments were performed independently in triplicates and data were expressed as Mean ± S.D. (n = 3), * p < 0.05 when compared to control group. # p < 0.05 when compared to CYL group. CYL: cyclophosphamide, SES: Sesamol.
